# Wide-Awake Surgery and Coronavirus Disease 2019: A Rural Experience

**DOI:** 10.1016/j.jhsg.2024.03.001

**Published:** 2024-04-01

**Authors:** Robert E. Van Demark, Troy D. Hollinsworth

**Affiliations:** ∗ University of South Dakota Sanford School of Medicine, University of South Dakota SSOM, Vermillion, SD; † University of South Dakota SSOM, Vermillion, SD

**Keywords:** Case volume, COVID-19, Hand surgery, Pandemic, WALANT

## Abstract

Surgeons across all subspecialties had to adapt to the Coronavirus disease 2019 pandemic to triage patients and steward hospital resources. Hand surgeons found themselves in a unique position to move some hospital-based procedures to a clinic-based setting, which has now impacted their postpandemic practices. Performing procedures in the clinic using the wide-awake local anesthesia no-tourniquet technique is interestingly similar to minor surgeries traditionally carried out in a general surgery clinic. By abstracting institutional case volumes from orthopedic, hand, and general surgery departments from 2019 to 2022, we identified trends that further support the potential for clinic-based procedures in hand surgery. This communication provides a foundation to compare cost and surgical indications for wide-awake local anesthesia across surgical disciplines.

During the Coronavirus disease 2019 (COVID-19) pandemic, hand surgery was among the many surgical specialties that had to adapt to unfamiliar circumstances. As hospital systems scrambled to allocate resources and staff to more critical patients, surgeons were forced to postpone nonurgent cases to later dates.^1^, ^2^, ^3^ Because most of the resource limitations were in the hospital-based operating room setting, some hand surgeons began to use the wide-awake local anesthesia no tourniquet (WALANT) technique in clinic-based procedure rooms.^4^^,^^5^ By implementing WALANT into hand surgery practices during the pandemic, some institutions have been able to recover from backed-up caseloads and improve resource utilization.^6^ As institutions report their experiences with WALANT, no studies to our knowledge have compared their hospital- and clinic-based case volumes before, during, and after the pandemic. By analyzing hand surgery numbers in comparison to both total joint arthroplasty and general surgery case volumes from a single institution, we were able to self-examine our response to the COVID-19 pandemic. Adding general surgery clinic procedure volumes allowed us to compare with another specialty performing procedures under wide-awake local anesthesia.

## Materials and Methods

Upon submission, the institutional review board determined that the study did not meet the criteria for human subjects’ research. Deidentified hospital and clinical case numbers were collected from a single institution from 2019 to 2022. The institutional database was queried for hospital-based case volumes in the following categories: all hospital procedures, all orthopedic surgery, hand surgery, total shoulder arthroplasty, total knee arthroplasty, total hip arthroplasty, and all general surgery cases. The institutional database was also queried for clinic-based case volumes for hand surgery and general surgery. Case numbers were analyzed by year (2019–2022) and April of each year (2019–2022). Hand surgery trauma cases from April 2020 were also counted and categorized by surgery type. April 2020 was considered the “lockdown month” of the COVID-19 pandemic in the institution’s region.

## Results

### All hospital surgery cases

When including all hospital cases from 2019 to 2022, the fewest were completed in 2020 (*n* = 21,968), and most were completed in 2021 (*n* = 25,961; Table). For the month of April, the fewest cases were in April 2019 (*n* = 763), and the most were in April 2021 (*n* = 2,200; Table). Hospital case data were not accessible for 2019.

**Table:** All Hospital, General Surgery, and Orthopedic Surgery Case Data

Department Year	All Hospital Surgical Specialties	General Surgery		Orthopedic Surgery					
	Hospital	Hospital	Clinic—Procedure	All Hospital	TSA∗	THA†	TKA‡	Hand (Hospital)	Hand (Clinic)
2019	25,010	5,486	696	7,893	175	463	762	426	178
2020	21,968	4,662	650	7,066	132	389	620	591	187
2021	25,961	5,288	560	8,124	214	519	836	659	224
2022	23,896	5,141	598	6,279	103	247	435	503	470
April-2019		459	70						
April-2020	763	232	32	184					0
April-2021	2,200	456	46	686					17
April-2022	1,903	408	49	479					41

∗ Total shoulder arthroplasty.

† Total hip arthroplasty.

‡ Total knee arthroplasty.

### All orthopedic surgery cases

For orthopedic surgery cases from 2019 to 2022, the fewest were completed in 2020 (*n* = 7,066), and the most were completed in 2021 (*n* = 8,124; Table). The same trend followed for the month of April of each year, showing the fewest orthopedic cases in April 2020 (*n* = 184), and the most in April 2021 (*n* = 686; Table). Hospital case data were not accessible for 2019.

### Total shoulder, hip, and knee arthroplasty cases

Total joint arthroplasty cases from 2019 to 2022 were also reviewed (Table). For total shoulder arthroplasty, 2022 had the fewest (*n* = 103), and 2021 had the most (*n* = 214). Total hip arthroplasty had the fewest cases in 2022 (*n* = 247), and the most in 2021 (*n* = 519). Total knee arthroplasty had the fewest cases in 2019 (*n* = 426), and the most in 2021 (*n* = 659). Monthly case totals were not accessible for total joint arthroplasty.

### Hand surgery cases

Hand surgery cases were recorded for both hospital- and clinic-based surgeries (Table). The year with the fewest hospital-based hand surgeries was 2019 (*n* = 426), compared with the most in 2021 (*n* = 659). For clinic-based hand surgeries, the fewest were completed in 2019 (*n* = 178), compared with the most in 2022 (*n* = 470). April-only data were only accessible for clinic-based hand surgeries, showing the fewest cases in 2020 (*n* = 0), and the most in 2022 (*n* = 41).

In April 2019, 20 hospital-based orthopedic hand surgeries were completed (Fig.). The three most common indications were distal radius fracture (*n* = 8), irrigation and debridement (*n* = 5), and extensor tendon repair (*n* = 3).

**Figure:**
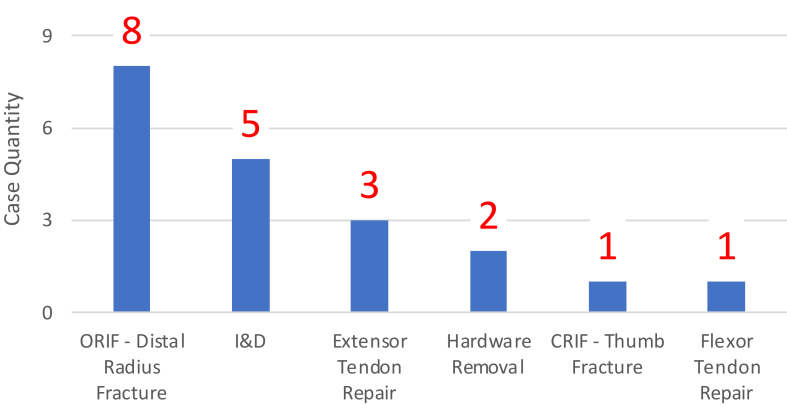
Hand trauma cases from “Lockdown Month” April 2020. Graph representing the breakdown of surgical indications for hand surgery during the “Lockdown Month” of April 2020. Twenty total cases were performed by the hand surgery department, with open reduction internal fixation of distal radius fractures being the most frequent indication at eight cases.

### General surgery cases

General surgery case numbers were also recorded in both the hospital-based and clinic-based settings for 2019–2022 (Table). The fewest general surgery hospital cases were completed in 2020 (*n* = 4,662), and the most in 2019 (*n* = 5,486). For the month of April of each year, hospital-based general surgery cases were the fewest in April 2020 (*n* = 232), and the most in April 2019 (*n* = 459).

For clinic-based general surgery procedures, the fewest were completed in 2021 (*n* = 560), and the most were completed in 2019 (*n* = 696). When looking at April only for general surgery clinic procedures, the fewest were in April 2020 (*n* = 32), and the most were in April 2019 (*n* = 70).

## Discussion

Overall, there is no question that the year 2020 came with obstacles for surgeons to complete cases. Resource and staff management appropriately prioritized the more critical patients in communities, but with fewer operating rooms available, opportunities for clinic-based procedures became of increasing interest. In April 2020, our hospital administration told the orthopedic hand surgery department that no elective cases could be performed in hospital- or clinic-based procedure rooms. This resulted in zero cases being completed in the clinic, with only 20 “nonelective” cases being performed during that month (Fig.). Seeing that other practices in the community (such as private dental and dermatology offices) did not receive the same restrictions, we saw the value of investigating whether hand surgery was differentially affected compared with general surgery, who performs both hospital- and clinic-based surgeries.

There is no shortage of literature explaining institutional experiences with WALANT techniques for the hand surgeon, but a few have compared the impact between surgical specialties.^2^ Levin et al^2^ reported that orthopedic surgery, ophthalmology, and gynecology were most affected, whereas cardiothoracic surgery, pediatric surgery, and general surgery were least affected by the pandemic. One author studied the overall impact of elective general surgery, revealing an overall trend toward outpatient, same-day surgery when possible.^7^ This brought about the question: how did clinic-based general surgery procedures get affected by the pandemic? Knowing that hemorrhoid banding, skin lesion resection, biopsies, and lipoma resections are often completed in the general surgeon’s clinic under local anesthesia, we thought it would be interesting to see if the pandemic affected these arguably “less urgent” procedures. The argument of decreased risk with local anesthesia over general anesthesia has been studied well, showing that patient outcomes are at least equal to general anesthesia, but with significantly lower costs.^8^

Looking at the results of this study, the general trend in the hospital was a decline in cases in 2020, with a rebound in 2021 with more cases than in 2019 (Table). This trend was consistent for total joint arthroplasty and orthopedic surgery as a whole. Of note, the decrease in total joint arthroplasty in 2022 was most likely because of some surgeons leaving the institution in 2022. April 2020 was consistently lower for both general surgery and hand surgery clinical procedures but did not appear to affect the yearly totals for either group. Interestingly, the number of hand clinic procedures only increased after 2020 (*n* = 187), totaling 470 cases in 2021. This trend should be correlated with the yearly hospital case data for hand surgery, which decreased overall from 2020 (*n* = 591) to 503 cases in 2022. The increased utilization of WALANT for these cases will be the focus of future studies.

This article briefly communicates how one institution compared the impact of the pandemic on clinic-based procedures performed under local anesthesia for patients without the use of sedation. Although this article has limited clinical implications, it draws attention to what can be learned from other surgical specialties. For example, seeing that general surgery has long-completed lipoma resections under local anesthesia in the clinic without issue, one can lower the threshold to complete more low-risk hand procedures in the clinic. The hand surgeon should continue to be attentive to techniques that improve the patient experience while preserving hospital resources.^4^^,^^6^

## Conflicts of Interest

No benefits in any form have been received or will be received related directly to this article.
